# Step by step full mouth rehabilitation of a class III edentulous patient by implant‐supported prosthesis: A case report

**DOI:** 10.1002/ccr3.1581

**Published:** 2018-05-15

**Authors:** Mahnaz Arshad, Gholamreza Shirani, Kamran Rasouli

**Affiliations:** ^1^ Dental Research Center Dentistry Research Institute Tehran University of Medical Sciences Tehran Iran; ^2^ Department of Prosthodontics School of Dentistry International Campus Tehran University of Medical Sciences Tehran Iran; ^3^ Department of Oral and Maxillofacial Surgery School of Dentistry Tehran University of Medical Sciences Tehran Iran; ^4^ International Campus Tehran University of Medical Sciences Tehran Iran

**Keywords:** dental implants, dental restorations, edentulism, orthognathic surgery

## Abstract

Full mouth rehabilitation by dental implants is complex in class III patients. Prediction of the outcome of orthognathic surgery in edentulous patients with no dental index or record is challenging. This report describes rehabilitation of an edentulous patient by placement of dental implants after prediction of the outcome of orthognathic correction.

## 1 INTRODUCTION

Some fully edentulous patients present with significant skeletal jaw malformations such as severe class III or II malocclusion with disproportionate facial structures, unfavorable maxillomandibular relationship, and asymmetric face. These discrepancies cause functional and esthetic problems for patients. The condition becomes worse if accompanied by congenital anomalies such as amelogenesis imperfecta.^1^ To achieve a good balance between the function and esthetics in patients with severe class III malocclusion, a step by step treatment process should include preprosthetic surgical procedures (bone grafting, orthognathic surgery, and implant placement) followed by prosthetic rehabilitation.^2^, ^3^

Herein, we present an edentulous class III patient who underwent a modified Epker prediction tracing, orthognathic surgery, and simultaneous bone grafting from the iliac crest. Full mouth rehabilitation was then performed using implant‐supported restorations.

## 2 CASE REPORT

**Figure 1:**
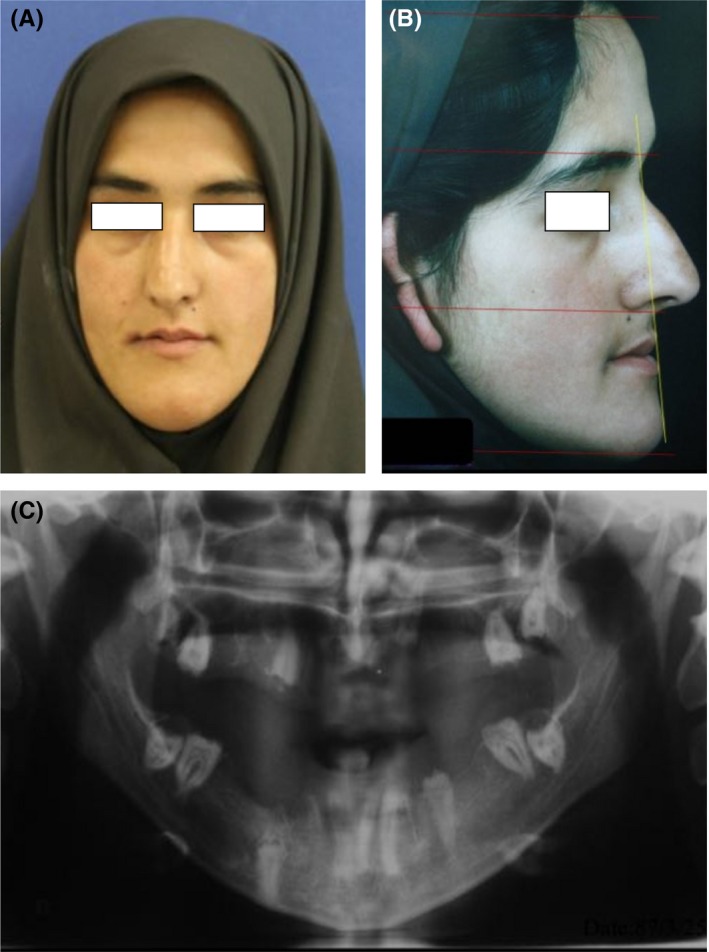
A, Preoperative frontal view. B, Preoperative lateral view. C, Preoperative panoramic view

In 2012, a 22‐year‐old edentulous female patient was referred to the Oral and Maxillofacial Surgery Department of Tehran University of Medical Sciences. She had severe class III malocclusion and facial deformity, which included nasal and mandibular deviation to the right. After taking a comprehensive medical and dental history, we noticed that the patient had hypodontia with several impacted teeth due to amelogenesis imperfecta (Figure 1).

**Figure 2:**
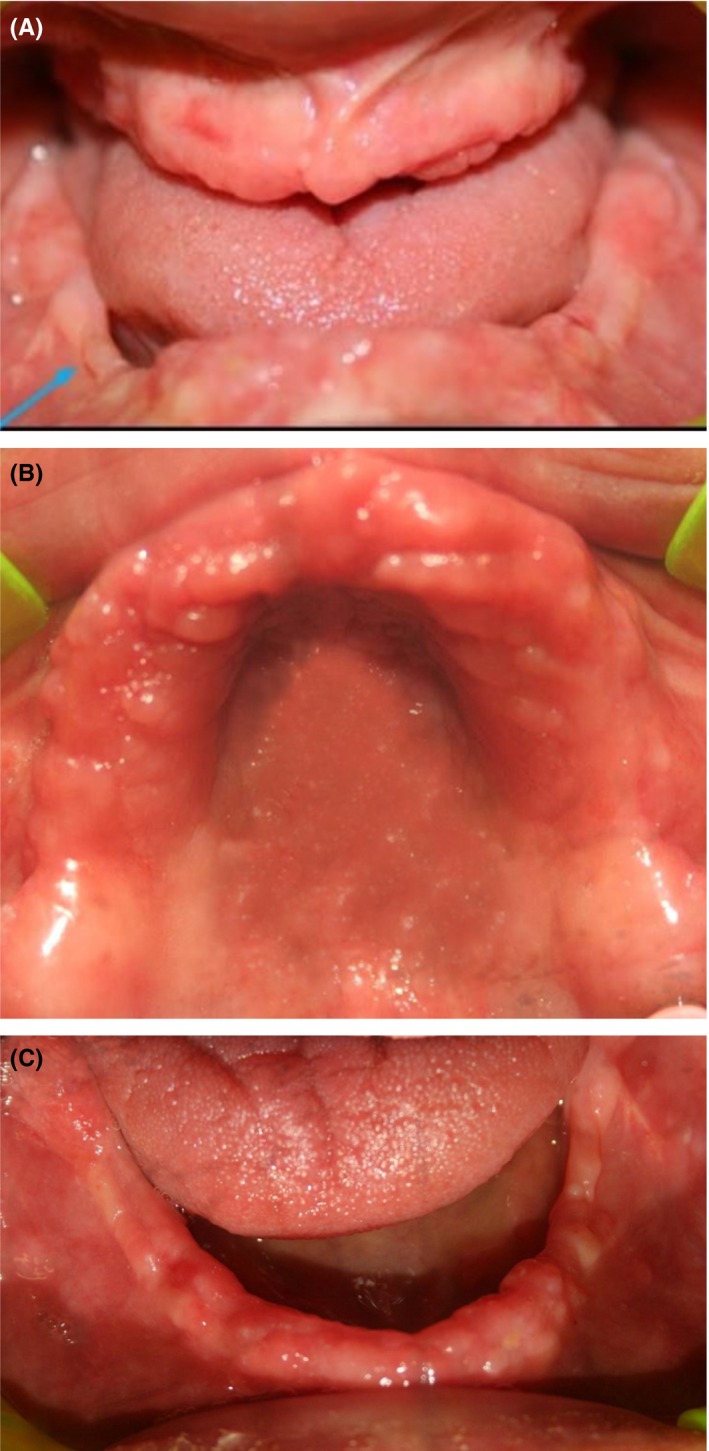
A, Intraoral frontal view showing uneven and knife‐edge alveolar ridge; B, Intraoral maxillary occlusal view; C, Intraoral mandibular occlusal view

Intra‐oral clinical examination revealed horizontal discrepancy of alveolar ridge, knife‐edge mandibular alveolar ridge, uneven alveolar ridge, and a deep palate (Figure 2).

Routine radiographic examinations consisting of panoramic radiography and lateral cephalometry were requested. The cephalogram exhibited anterior‐posterior discrepancy of the jaw in the horizontal plane and a pseudo‐long face.

Following consultation with a prosthodontist, cosmetic surgery was scheduled for the patient to correct her long face, mid‐face deficiency, jaw deviation to the right, class III discrepancy, and alveolar ridge deficiency followed by dental implant placement and full mouth prosthetic rehabilitation. After consultation with an orthodontist, it was found that forced eruption of the impacted teeth was not possible; thus, the teeth were extracted under local anesthesia.

**Figure 3:**
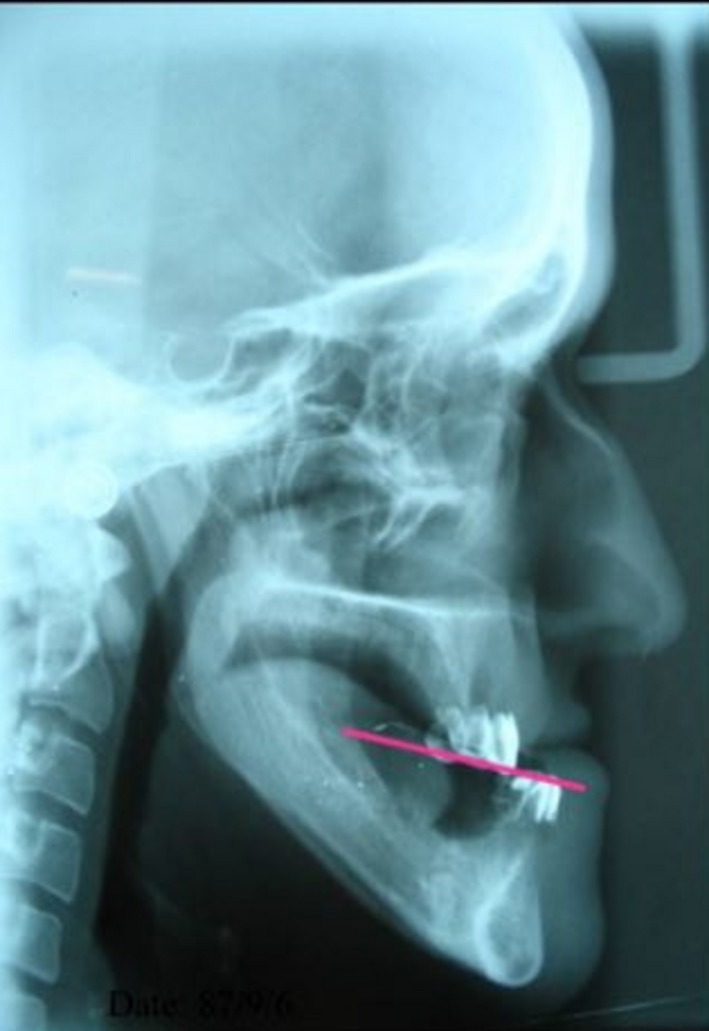
Preoperative panoramic view

Primary impressions were made using irreversible hydrocolloid impression material (Kimica, Tokyo, Japan). Special trays were fabricated, and final impression was made by zinc oxide eugenol (Wuhan Xingzhengshun, Hubei, China). Then, the occlusal rims were made to record the inter‐arch relationship, and the casts were mounted in a semi‐adjustable articulator (Dentatus, New York, USA) in centric relation. The teeth were arranged in class III occlusion, and then they were coated with barium sulfate (Foshan Xinmei Chemical, Guangdong, China) to make them opaque for easy detection of the occlusal line for Epker cephalometric prediction tracing (Figure 3).

**Figure 4:**
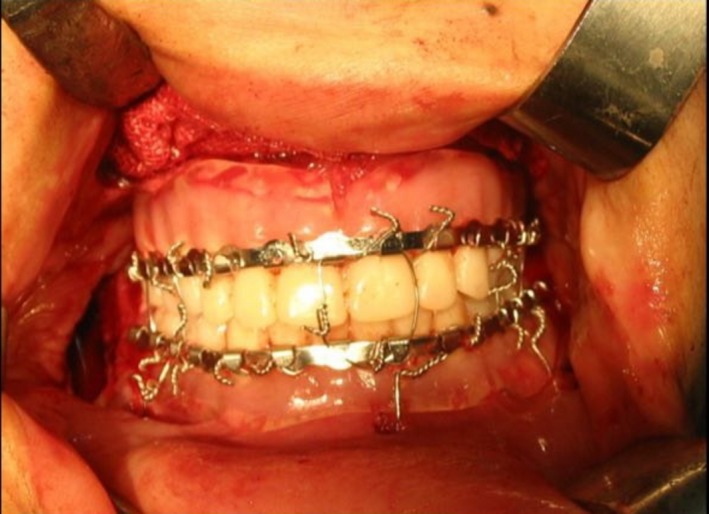
Bimaxillary fixation with a surgical stent

The Epker prediction tracing was performed for preoperative assessments, which included analysis of the maxillary retrognathism, mandibular prognathism, and excess skeletal vertical growth. Sagittal split ramus osteotomy and LeFort I osteotomy were scheduled. According to the data obtained from the lateral cephalogram of the patient and clinical examinations (zygomatic regions, paranasal area, nasolabial, and gonial angles, SNA, SNB, and ANB angles), the surgeon planned a mandibular setback procedure by 7 mm and repositioning of the maxilla by 2 mm superiorly and 5 mm anteriorly on surgical casts. Then, the casts were mounted in this new position and the jaw relations were determined. Posterior teeth were arranged in new articulation and the final dentures were fabricated. The surgeon used these dentures for the edentulous patient as the final surgical splint. Mandibular cast and denture setback by 7 mm relative to its current position and an intermediate stent was fabricated using auto‐polymerizing acrylic resin (Kulzer, Newbury, UK). Then, dentures were fabricated as splint and after polishing, several holes were drilled in the base of denture, and the arch bar was bonded to it. Circummandibular wires for the mandible and palatal screws were used to fix the splints to the corresponding jaws. The surgical procedure of the maxilla and mandible was performed using these splints (Figure 4).

Alveolar ridge augmentation was performed by harvesting a graft from the iliac crest. The graft was placed upon a surgical stent and fixed to the external maxillary surfaces with a screw and plate.

**Figure 5:**
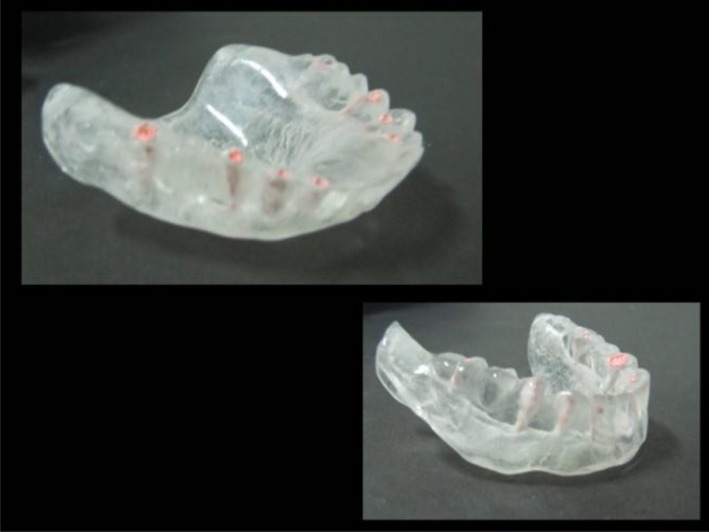
Radiographic stent for implant placement

After 2 weeks, temporary complete dentures were delivered to the patient. They were adjusted using tissue conditioner (Kerr, USA). Six months later, an impression was made and a radiographic stent was fabricated for implant placement. To determine the proper location for placement of implants, gutta‐percha points (VDW, Munich, Germany) were placed at the center of desired teeth in ideal direction of implant, and the outer surfaces of the teeth were coated with barium sulfate (Foshan Xinmei Chemical, Guangdong, China) (Figure 5).

**Figure 6:**
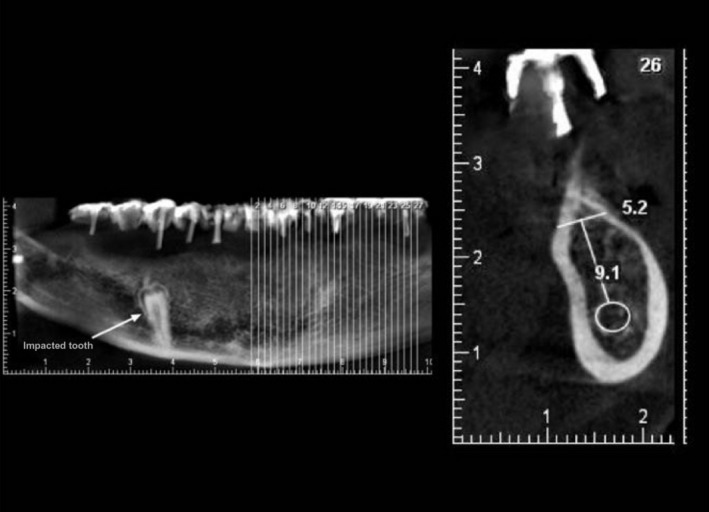
CBCT scan of alveolar ridge with radiographic stent

The patient underwent cone beam computed tomography with stent to determine the proper site of implant placement (Figure 6). Proper diagonal length and angle of 16 implants (Dentium, Implantium, Korea, Seoul), eight in each jaw, were determined by stent according to the available bone volume. Implants were inserted using a surgical stent. The temporary complete dentures of patient were relined and delivered to the patient. Three months later, the implants were uncovered, and healing abutments were tightened. Primary impression of the healing was made by alginate (Kimica, Tokyo, Japan). Primary cast was poured. After fabrication of special tray, final impression was made using the open tray technique following splinting of impression copings using polyvinyl siloxane (Zhengzhou Huaer, Henan, China). The final cast was fabricated. Impression verification jig was fabricated in a dental laboratory and was tried in patient's mouth to confirm the accuracy of impression. Next, the record base and wax rims were fabricated. The jaw relations were recorded by rims, and after that the teeth were arranged. The teeth set‐up was checked in the mouth. A putty index was made from the arranged teeth and accordingly, proper abutment angle and gingival height were determined.

**Figure 7:**
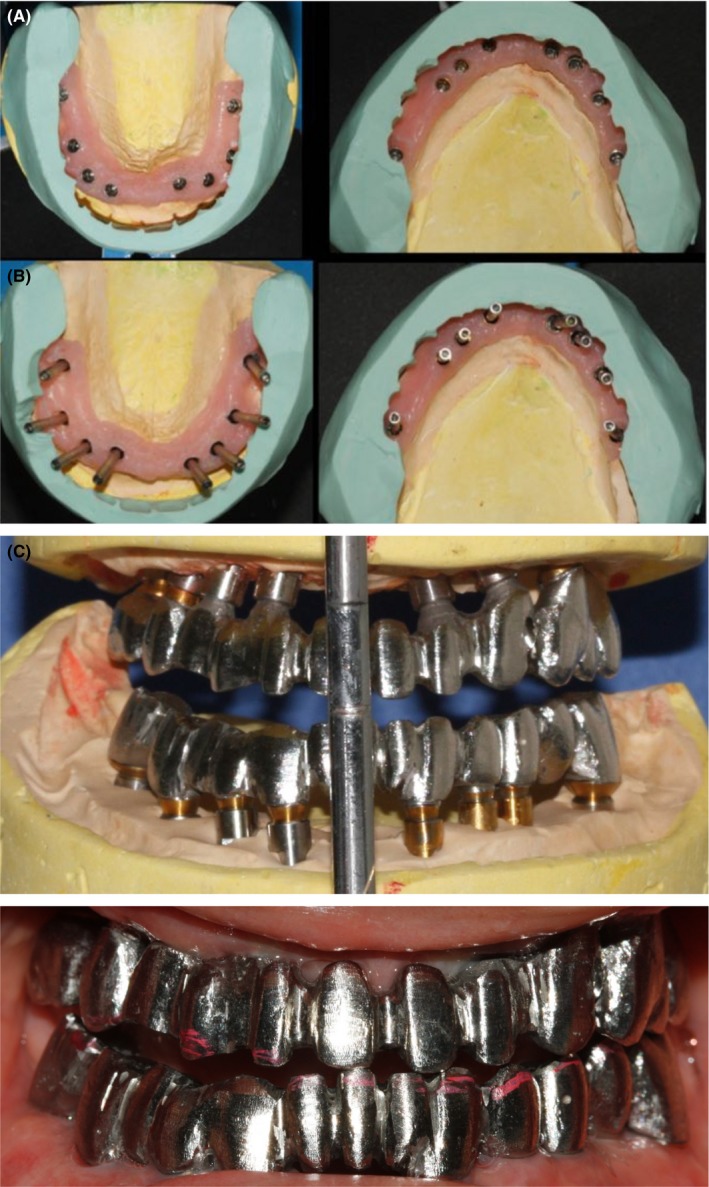
A, Putty index for abutment selection; B, framework on the cast; C, intraoral view of framework

**Figure 8:**
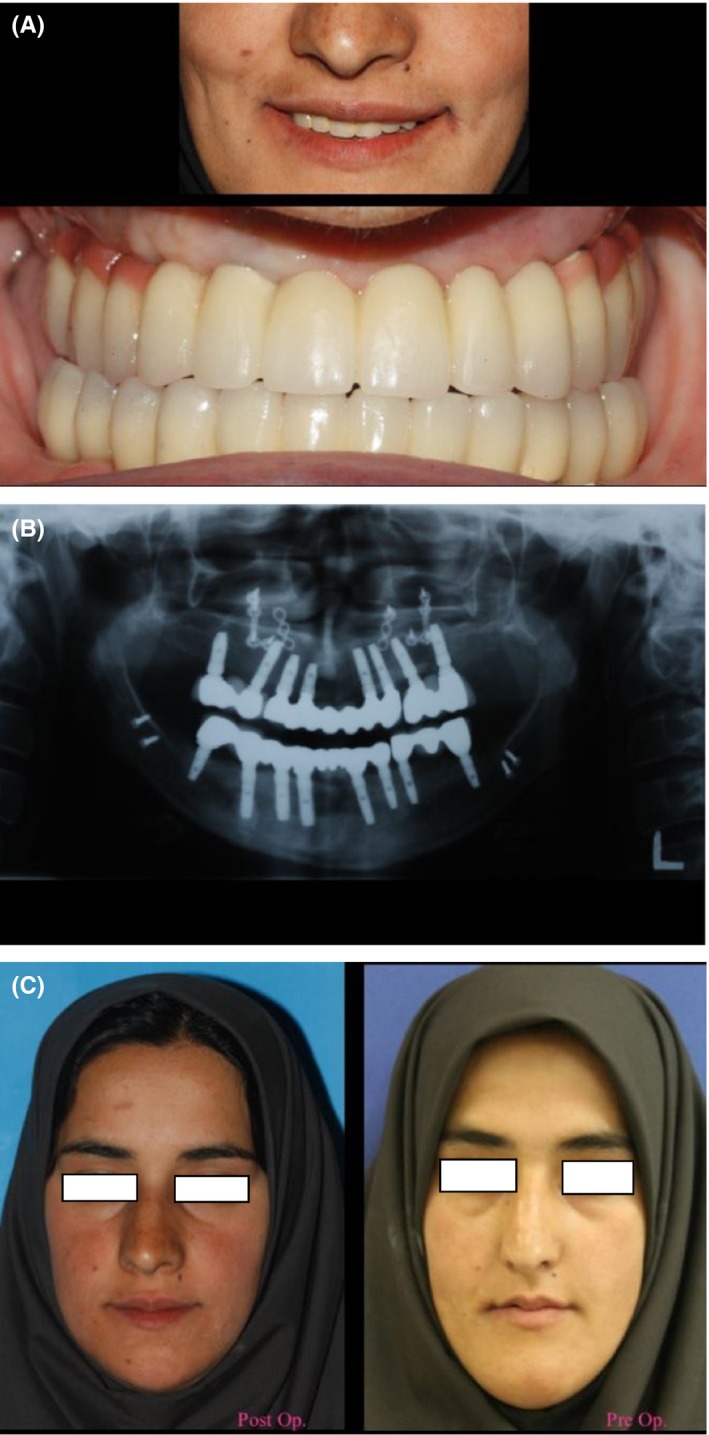
Final view of full mouth rehabilitation. A, Frontal intraoral view. B, Panoramic view after implant placement. C, Preoperative and postoperative frontal view of patient

The three‐part frames (Degubond, Dentsply sirona prosthetics, U.S.A, Pennsylvania) for the maxilla and mandible were fabricated. Frames were tried in the mouth, and the fit of frames was checked radiographically (Figure 7). Porcelain crowns with A2 shade were fabricated. Implant‐supported crowns were cemented with a temporary cement (Kerr, Toronto, Canada) (Figure 8). The patient was fully satisfied with the results at the six‐year follow‐up.

## 3 DISCUSSION

Full mouth rehabilitation of edentulous patients with dental implants is clinically challenging because of alveolar ridge resorption, inadequate residual bone, and increased interarch space. In severe atrophy of the jaws and severely resorbed alveolar ridges, bone augmentation or intraoral bone grafting is performed to enhance the alveolar ridge volume for placement of endosseous dental implants.^2^, ^3^ In other words, adequate bone volume is a prerequisite for placement of dental implants to ensure optimal function and esthetics. Many surgical procedures have been suggested for management of such patients including block grafting and lateral augmentation with or without guided bone regeneration to augment the ridge.^4^

One indication of orthognathic surgery is for fully or partially edentulous class III patients.^5^ Proper maxillomandibular relationship provides good support for the upper lip improves the patient profile and results in better occlusion.^6^

Cephalometric radiography plays an important role in planning of orthognathic surgery. Cephalometric prediction tracing for orthognathic surgery is important as it helps in selection of proper surgical approach and simulates the surgical procedure on a model. Changes in hard tissue directly affect the soft tissue shape and position. Cephalometric prediction tracing also determines the need for further surgical procedures. The Epker's orthodontic‐surgical cephalometric prediction tracing requires the determination of occlusal plane. Using this method, we determined the occlusal plane on a lateral cephalogram.^7^ Occlusal plane determination is an important factor in Epker's prediction. In edentulous patients, using radiopaque teeth or teeth covered with barium sulfate is helpful in occlusal plane determination on lateral cephalograms.

The most important factor in treatment planning for such complicated cases is to use a multidisciplinary approach and consult with specialists to achieve ideal results.

## 4 CONCLUSION

Full mouth rehabilitation of completely edentulous class III patients by implant placement, as in our patient, is challenging especially in the presence of maxillofacial deformities or inadequate volume of alveolar ridge. Complete denture used to be the most commonly suggested treatment plan for edentulous patients. Implant‐supported dentures can provide optimal masticatory function with fewer complications such as bone resorption.

## CONFLICT OF INTEREST

None declared.

## AUTHORSHIP

MA: involved in conception and design of the work, collected the data, drafted the manuscript, performed critical revision of the manuscript, and approved the final version to be published. GS: involved in conception and design of the work, collected the data, drafted the manuscript, performed critical revision of the manuscript, and approved the final version to be published. KA: involved in data collection, drafted the manuscript, performed critical revision of the manuscript, and approved the final version to be published.
